# CRISPR-Cas Systems: A Functional Perspective and Innovations

**DOI:** 10.3390/ijms26083645

**Published:** 2025-04-12

**Authors:** Carla Navarro, María P. Díaz, Pablo Duran, Ana Castro, Andrea Díaz, Clímaco Cano, Ana-Karina Carbonell-Zabaleta, Donny-Sabrith Solano-Jimenez, Diego Rivera-Porras, Julio César Contreras-Velásquez, Valmore Bermúdez

**Affiliations:** 1 Endocrine and Metabolic Diseases Research Center, School of Medicine, University of Zulia, Maracaibo 40001, Venezuela; mariadiazalbornoz@hotmail.com (M.P.D.); pabloduran1998@gmail.com (P.D.); anavaleriacastro@hotmail.com (A.C.); andreavalentinadiaz@hotmail.com (A.D.); antioxidante48@gmail.com (C.C.); 2 Universidad Simón Bolívar, Facultad de Ciencias de la Salud, Programa de Medicina, Barranquilla 080001, Colombia; ana.carbonell@unisimon.edu.co (A.-K.C.-Z.); donny.solano@unisimon.edu.co (D.-S.S.-J.); 3 Universidad de la Costa, Departamento de Productividad e Innovación, Barranquilla 080001, Atlántico, Colombia; drivera23@cuc.edu.co (D.R.-P.); jcontrer30@cuc.edu.co (J.C.C.-V.); 4 Universidad Simón Bolívar, Facultad de Ciencias de la Salud, Centro de Investigaciones en Ciencias de la Vida, Barranquilla 080001, Colombia

**Keywords:** CRISPR-Cas, gene editing, gene therapy

## Abstract

Adaptation is a fundamental tenet of evolutionary biology and is essential for the survival of all organisms, including prokaryotes. The evolution of clustered regularity exemplifies this principle of interspaced short palindromic repeats (CRISPR) and associated proteins (Cas), an adaptive immune system that confers resistance to viral infections. By integrating short segments of viral genomes into their own, bacteria and archaea develop a molecular memory that enables them to mount a rapid and targeted response upon subsequent viral challenges. The fortuitous discovery of this immune mechanism prompted many studies and introduced researchers to novel tools that could potentially be developed from CRISPR-Cas and become clinically relevant as biotechnology rapidly advances in this area. Thus, a deeper understanding of the underpinnings of CRISPR-Cas and its possible therapeutic applications is required. This review analyses the mechanism of action of the CRISPR-Cas systems in detail and summarises the advances in developing biotechnological tools based on CRISPR, opening the field for further research.

## 1. Introduction

CRISPR-Cas has emerged as the most reliable and efficient technique for genome editing in eukaryotic cells, employing nucleases as “molecular scissors” developed from the adaptive immune mechanism of prokaryotic organisms [1,2]. Current research has identified 6 types and 29 subtypes of CRISPR-Cas, all operating on the same principle of CRISPR RNA (crRNA) in the CRISPR-Cas9 experimental system to achieve specificity when applied to guide RNA (gRNA) [2].

CRISPR-Cas technologies have facilitated the elucidation of how alterations at specific loci impact the human epigenome and its regulatory mechanisms, enabling disease model development and the identification of novel components involved in cellular differentiation and reprogramming. These advancements have unveiled promising therapeutic avenues, including T-cell engineering for cancer immunotherapy and monogenic disorders treatment [3,4,5,6]. In the future, this technology is expected to fight infections such as HIV, although this area is still under investigation [7].

However, several key limitations have been identified for the clinical translation of CRISPR-Cas technologies, including (1) the risk of off-target editing, potentially affecting unintended genomic loci; (2) the activation of immune responses against various components of the system, including the delivery vector and the specific Cas protein employed; (3) challenges associated with the delivery of the gene-editing machinery and the precise modification of target cells; and (4) the difficulties inherent to in vivo delivery of the editing components. Additionally, ethical considerations regarding human genome modification have generated discussions with both pros and cons, highlighting the monetary cost, effectiveness, access to this resource, and the need to create regulating legislation [8,9,10]. Nevertheless, the rapid pace of development of this technology fuels the hope that in the future, genome editing through the CRISPR-Cas system is expected to be a first-line tool for the treatment of diseases that currently diminish the quality of life [5,6,11]. Therefore, this review aims to present the current information on CRISPR as a tool, emphasising a deep description of its functioning and applicability in the medical field.

## 2. Bacterial Adaptive Immunity: CRISPR-Cas Systems and Their Mechanisms of Action

**Figure 1:**
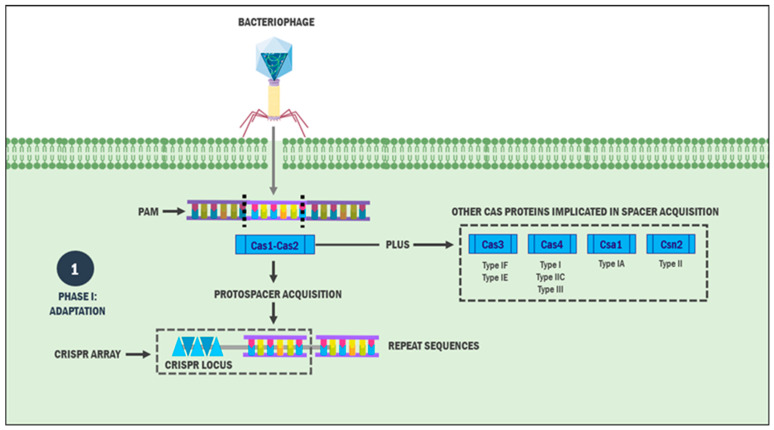
Mechanism of adaptation in CRISPR-Cas systems. After including foreign genetic material inside the bacterium, Cas1 and Cas2 proteins are responsible for recognising and excising a specific portion of it: the protospacer. Then, this is included within the CRISPR sequence to carry out the subsequent steps of bacterial adaptive immunity. Some CRISPR subtypes use other Cas proteins to acquire said protospacer. Abbreviation: PAM: Protospacer adjacent motive.

**Figure 2:**
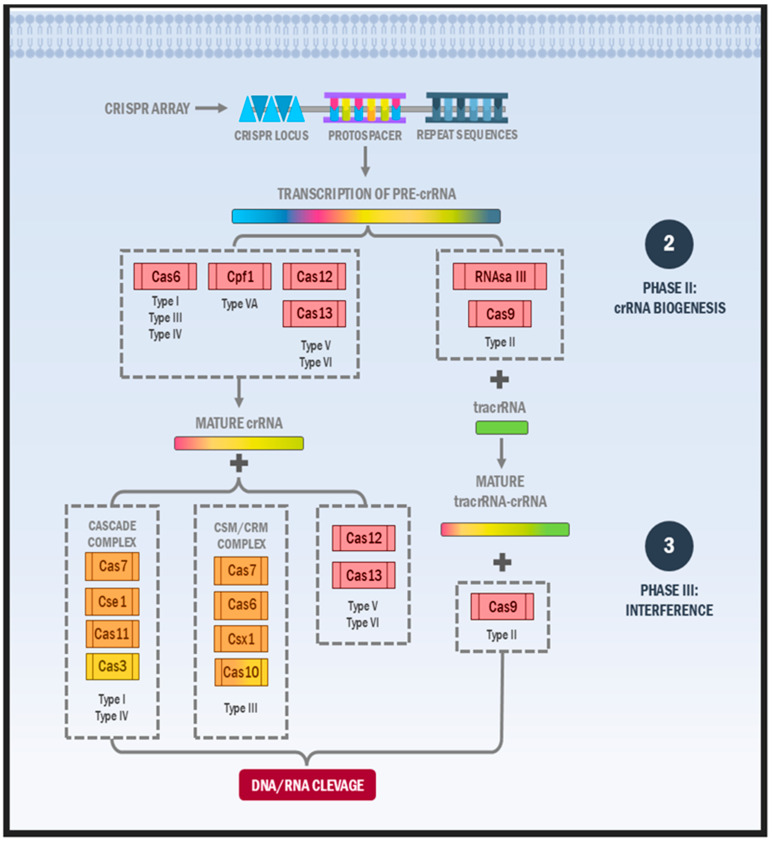
Mechanism of crRNA biogenesis and interference in CRISPR-Cas systems. The pre-crRNA transcription occurs from the CRISPR array. Subsequently, different Cas and non-Cas proteins (RNAase III) are responsible for the crRNA maturation. Then, the crRNA and certain specialised complexes and proteins are responsible for recognising and destroying foreign DNA and/or RNA. Abbreviations: DNA: deoxyribonucleic acid; RNA: ribonucleic acid.

CRISPR-Cas systems constitute an adaptive immune system in bacteria and archaea, operating through a three-phase mechanism: (a) adaptation, involving the acquisition of new spacers in the CRISPR locus derived from foreign genetic elements such as viral DNA/RNA, plasmids, and bacteriophages; (b) crRNA biogenesis, encompassing the transcription of the CRISPR locus into a precursor RNA and its subsequent processing by ribonucleases; and (c) nucleic acid interference, where mature crRNAs guide Cas enzymes to target and cleave homologous invading nucleic acids (e.g., viral DNA), leading to their degradation (Figure 1 and Figure 2) [12]. It is important to note that while these three stages are conserved across all CRISPR-Cas systems, the specific Cas proteins involved and the molecular details of each phase can vary significantly among microorganisms [13].

### 2.1. Phase I: Adaptation

The initial phase involves incorporating a sequence from the invading foreign genetic element (MGE), termed a protospacer (30 to 50 base pairs), into the CRISPR array or locus as a new spacer sequence adjacent to the leader sequence [14]. Protospacer selection is not random but is guided by protospacer adjacent motifs (PAMs), 2–5-nucleotide sequences that play a crucial role in discriminating the specific genetic material sequences necessary for subsequent processes [15].

Although the precise molecular mechanisms of MGE incorporation into the CRISPR array remain incompletely understood, this stage is universally executed across the systems by two metal-dependent nucleases, Cas1 and Cas2 [16,17]. In type I CRISPR systems, Cas1 binds to the PAM complementary sequence in either single-stranded DNA (ssDNA) or double-stranded DNA (dsDNA) form [17,18]. Specifically, Cas1 recognises the PAM sequence 5′-CTT-3′, positioning the protospacer’s phosphodiester bond within the Cas1 active site [19]. Studies have demonstrated that Cas1 and Cas2 form a structural complex responsible for protospacer acquisition and integration [20].

Investigations of type I CRISPR system adaptation in *E. coli* have revealed that the Cas1–Cas2 heterohexameric complex required for protospacer acquisition comprises two Cas1 dimers (Cas1a, Cas1a′, Cas1b, and Cas1b′) and a Cas2 dimer [19]. While Cas2’s catalytic site is dispensable, Cas1 is the main effector of this complex. However, Cas2 alterations interfere with a new protospacer acquisition in the CRISPR locus, suggesting that Cas2 possesses structural and mechanistic functions for this first phase [20]. Therefore, the hypothesis that both proteins are essential for acquiring and integrating new spacers into the CRISPR locus is plausible.

The type IE system of *E. coli* initiates spacer acquisition through PAM recognition and complementary sequence identification in dsDNA/ssDNA from the MGE via the Cas1a and Cas1a′ subunits [21]. The protospacer identified by the Cas1–Cas2 complex comprises 33 base pairs [19]. Furthermore, tyrosine residues (Tyr22) of the Cas1 and Cas1 subunits bind to the MGE, limiting the central duplex region of the protospacer to 23 base pairs and leaving free ends of 5 base pairs on each side, and the subsequent dsDNA cleavage forms ssDNA strands [19,22,23]. Cas2 stabilises this process through interactions between the Cas1–Cas2 complex’s arginine clamp and the protospacer phosphodiester backbone’s phosphate groups. Subsequently, each Cas1 dimer monomer threads both 3′ ends of the ssDNA strand through an arginine-rich channel, positioning OH groups at each 3′ end [19].

Furthermore, the Cas1–Cas2 complex exhibits integrase activity, enabling protospacer acquisition from the MGE into the CRISPR locus [20,24,25]. Evidence suggests that this process occurs in two distinct steps. Initially, the 3′OH group at each protospacer end catalyses transesterification reactions through nucleophilic attacks on the CRISPR locus negative strand, forming a branched intermediate that enables protospacer binding to the strand’s 5′ end. This attack occurs sequentially: first at the leader guanosine-rich repeat junction of the top strand, then at the guanine of the repeat-spacer junction of the bottom strand.

In the second step, after forming the covalent bond between the repeat matrix strand and protospacer, the protospacer attacks the junction between the first CRISPR repeat and the leader sequence of the positive strand [25]. The protospacer is integrated into the CRISPR locus, becoming a new spacer in the first CRISPR repeat. As a result, a new spacer derived from the MGE is acquired [25,26]. At the end of this process, DNA polymerases and other, yet unidentified, ligation systems mediate the production of a new copy of the original repeat, positioning it at the leader end of the CRISPR locus, thus acting as a recipient structure for the addition of new spacers. However, not all CRISPR systems have verified this process [25,27,28].

The host integration factor (IHF) is involved in the machinery of acquiring new spacers (R[MPDA1]). In addition to having a binding site for the Cas1–Cas2 complex, the CRISPR locus also contains a binding site for IHF [29]. IHF induces CRISPR DNA bending, which could bring the leader region closer to the first repeat. In turn, this DNA bending is likely to facilitate the proximity of the Cas1–Cas2 complex to the protospacer and the leader repeat, allowing nucleophilic attacks by the 3′-OH ends of the spacer fragment at the leader–repeat junction, thus facilitating the process of integrating new spacers [29].

Because of this, it is suggested that the proximity of these regions, in conjunction with Cas proteins, leads to the integration of the protospacer into the leader region of the CRISPR locus. Notably, IHF is not found in all bacteria, but is primarily in Gram-negative bacteria [30]. Additionally, studies have shown that the RecBCD exonuclease complex can contribute to the adaptation process by cleaving dsDNA for subsequent recognition of ssDNA by Cas1–Cas2 [30,31,32].

Although the aforementioned mechanisms form the basis of the adaptation–acquisition process, they are not reproducible in all CRISPR systems. For instance, the type IB system of *Haloarcula hispanica* demonstrates direct Cas4 involvement in spacer acquisition [33], whereas in the type IA system of Thermoproteus tenax, an effector complex composed of Cas1–Cas2, Cas4, and Csa1 was formed [34]. Furthermore, the fusion of Cas2 with Cas3 has been observed in type IF systems. In a *Pectobacterium atrosepticum* model, Cas1 interacted with the hybrid fusion protein Cas2–Cas3, promoting the hypothesis that Cas3 acts in nucleic acid interference and spacer acquisition [35].

In type II CRISPR-Cas systems, Cas9 mediates spacer selection by initially recognising the PAM sequence in double-stranded DNA (dsDNA) and, subsequently, Cas1, Cas2, and Csn2 recruiting for new spacer integration into the CRISPR locus [36]. Csn2, encoded by all type IIA systems, plays a vital role in adaptation by either stabilising dsDNA cleavage during spacer integration [37,38] or forming a ring-shaped tetrameric complex with a positively charged central cavity for DNA fragment binding [38,39]. Studies have also demonstrated the CRISPR locus trans-activator’s involvement in type II system protospacer acquisition [40].

The type II-C system of *Campylobacter jejuni* employs a phage-encoded protein resembling Cas4 during the adaptation stage [41]. Cas4, from the type I and III systems seen in some bacteria and archaea, forms fusion products with Cas1–Cas2, suggesting a functional complex capable of mediating spacer acquisition [42,43]. Cas4’s role may relate to its RecB-like exonuclease domain, which cleaves ssDNA bidirectionally (5′-3′ or 3′-5′) [44,45]. In vitro studies of the type IIIB system of *Marinomonas mediterranea* revealed that Cas1 fuses with a reverse transcriptase to acquire foreign RNA protospacers, which are subsequently reverse-transcribed into DNA spacers [46]. The subtype VC system contains only a Cas1 homolog and the C2C3 effector protein involved in this phase [47,48].

An alternative mechanism, *primed spacer acquisition*, counteracts MGE escape mediated by PAM mutations [49]. This process functions as a positive feedback loop, accelerating the incorporation of new protospacers into the CRISPR locus, predominantly from plasmid-derived MGEs [50]. Primed spacer acquisition is particularly prevalent in type I CRISPR-Cas subtypes. In subtype IE, this mechanism necessitates the involvement of Cas1, Cas2, Cas3, and a ribonucleoprotein complex comprising crRNA and multiple Cas proteins called the cascade complex [51]. In *E. coli* models, primed spacer acquisition preferentially targets the same strand as the original primed spacer, suggesting a strand bias in this process [52].

Unlike the type IE system, the type IF system incorporates new spacers targeting either strand of the invading MGE protospacer. This process involves a Cas1:Cas2–3 complex interacting with Csy for subsequent protospacer integration into the CRISPR locus [49]. Studies of type IF systems in L. pneumophila have demonstrated that many CRISPR systems acquire spacers derived from plasmids through priming processes via horizontal gene transfer [53]. The universal adaptation–acquisition process and priming mechanism are crucial for developing a CRISPR locus capable of recognising and destroying homologous invaders.

### 2.2. Phase II: crRNA Biogenesis

Following spacer acquisition in the CRISPR locus, the crRNA biogenesis phase begins with co-transcribing the new spacer alongside existing spacers and/or repeats, producing a long pre-crRNA [54]. Maturation requires various system-specific reactions. Class I systems (types I and III) employ specific RNP complexes: the cascade complex (type I), Csm proteins (type IIIA), and Cmr proteins (type IIIB). In contrast, Class 2 systems (types II, V, and VI) utilise Cas9 (type II), Cpf1 or associated proteins (type V), and Cas13 (type VI) [43,55].

In Class I systems, the primary effector of pre-crRNA maturation is the Cas6 endoribonuclease, a member of the Repeat-Associated Mysterious Proteins (RAMPs). As a component of various RNP complexes, Cas6 cleaves pre-crRNA within the repeat sequence [56,57]. Typically, cleavage occurs at the stem-loop structure’s base formed by the CRISPR palindromic repeat, specifically at the phosphodiester bond eight nucleotides upstream of the spacer sequence’s 5′ end, yielding mature crRNA of approximately eight nucleotides [58,59,60]. Some type I system subtypes exhibit variations in this process. In subtypes IA and IC, SsCas6 (a Cas6 homolog) and Cas5d (a Cas5 variant) replace Cas6 activity [61,62]. The latter protein is considered a RAMP, and the resulting crRNA comprises 11 nucleotides in the 5′ direction [63,64].

In type IE and IF systems, where Cas6 variants (Cas6e and Cas6f, respectively) are components of the cascade complex, the mature crRNA remains bound to this complex via its 3′ end for subsequent recognition and destruction of the invading MGE [60]. Type III systems employ similar mechanisms but utilise protein analogues (Csm and Crm) rather than the cascade complex. Additionally, their mature crRNA repeats do not form stem-loop structures, and some subtypes further process the crRNA by removing repeated sequences at the 3′ end through undefined nucleases [59,65,66,67]. Type IV CRISPR systems belonging to Class I contain Cas6-like proteins and cascade complex analogues, suggesting similar mature crRNA activation mechanisms [68].

In contrast to Class 1, Class 2 CRISPR systems do not work with RNP complexes or Cas6 for mature crRNA biogenesis but with large individual proteins like Cas9 and other non-Cas proteins such as RNase III [55]. Similarly, for this stage, type II systems require the presence of small tracrRNA molecules for pre-crRNA recognition. The anti-repeat sequence of tracrRNA allows its pairing with pre-crRNA, leading to the formation of a tracrRNA–pre-crRNA complex characterised by being a double-stranded RNA (dsRNA) [69].

Cas9 is a molecular anchor, stabilising the pairing process [69]. Subsequently, RNase III recognises and cleaves the tracrRNA–pre-crRNA complex to generate mature tracrRNA-crRNA [14]. Like type III systems, undefined nucleases trim the crRNA at either the 5′ or 3′ end, while tracrRNA undergoes cleavage exclusively at the 5′ end [69,70,71]. Both tracrRNA and crRNA are essential for the interference stage in type II systems [72].

On the other hand, Fonfara et al. [73] determined that the type VA system of *Francisella novicida* uses the dual nuclease Cpf1 for crRNA biogenesis. This protein cleaves a hairpin structure of the pre-crRNA, producing intermediate crRNAs that, through the aforementioned mechanisms, are further shortened to produce mature crRNAs. Similarly, it has been observed that in type V and VI systems, Cas12a and Cas13 proteins can mediate the crRNA maturation process without the need for the co-expression of tracrRNA and RNase III [47,73,74].

Studies have revealed that Cas13 homologs (Cas13a, Cas13b, Cas13c, and Cas13d) perform similar functions in mature crRNA formation within type VI systems [74]. Following maturation, crRNAs from various CRISPR systems bind to their respective Cas machinery, directing the recognition and subsequent destruction of homologous foreign MGE [12].

### 2.3. Phase III: Nucleic Acid Interference

The interference stage involves mature crRNA–Cas protein complexes identifying homologous foreign nucleic acids—viral material or plasmid sequences—through complementary base pairing in the presence of PAM sequences (in most systems). This recognition leads to target genetic sequence degradation by Cas protein nuclease domains [75,76]. Interference mechanisms vary among CRISPR systems: types I, III, and IV employ RNP complexes for foreign MGE degradation, while types II, V, and VI utilise single Cas proteins [76,77].

To prevent self-targeting, types I, II, IV, and V systems employ PAM sequence recognition [60,78], while most type III and IV systems discriminate self from non-self through 5′ tags of mature crRNA and species-specific protospacers (PFS) analogous to PAMs, respectively [79,80]. Regarding type I systems, after scanning the invading DNA, base pairing proceeds between the invader and the crRNA, specifically between the 6–8 nucleotide “seed” region of the spacer and the complementary protospacer, followed by the complete pairing of both elements, resulting in the displacement of the target strand and R-loop structure formation [65].

The scanning process depends on crRNA–cascade complex interaction [81,82,83]. Cse1 mediates PAM recognition through interactions between its flexible L1 loop and 3-nucleotide AAG PAM sequences located in Cse1’s N-terminal domain [83,84]. PAM recognition requires specific structural elements in the Cse1 subunit, including glycine loops, a lysine finger, and a glutamine wedge [85].

Following crRNA cascade complex binding to dsDNA, the complex destabilises the dsDNA, enabling crRNA hybridisation and base pairing with the seed region at the spacer’s 5′ end (nucleotides 1–5 and 7–8) [86,87]. This process generates a complete R-loop, stabilised by the cascade complex’s Cse2 (or Cas11) subunit homodimer. These subunits interact with Cse1 in the complex’s lower portion and Cas6e in the upper portion [72,88,89].

Subsequently, the complex undergoes conformational changes in both small and large subunits, triggering the recruitment of Cas3 by the Cse1 subunit, allowing its binding to the cascade [81]. Cas3 initially performs an endonucleolytic cut on the non-target strand within a 7–11 region comprising a substantial portion of the R-loop. This cleavage enables Cas3’s exonucleolytic degradation of the target DNA [90,91,92]. Since this reaction can produce intermediate degradation products, host nucleases or Cas3 mediate final complete degradation in an ATP-dependent manner [93,94].

While the type I system interference mechanism is primarily based on *E. coli* IE, subtypes exhibit variations. For example, type I Fv does not contain Cse1, but Cas5fv has an additional domain that allows it to fulfil its roles [95], while in other variations of type IF, Cas2–Cas3 binding is observed. In turn, in subtypes IA, IB, and ID, the properties of Cas3 are divided into two distinct proteins: Cas3′ (helicase activity domain) and Cas3″ (nuclease activity domain) [96].

The type II CRISPR system employs the tracrRNA–crRNA complex to guide Cas9 in cleaving target homologous MGEs [72]. Viral DNA strand interference requires recognising a 5′-NGGNG-3′ PAM sequence downstream near the protospacer’s 3′ end [97,98]. The interaction and subsequent Watson–Crick base pairing of the 20 base pair sequences between the invading DNA and the guide crRNA protospacer, particularly within a 10–12 nucleotide seed sequence at the target sequence’s distal end, leads to DNA:crRNA heteroduplex formation [72,99].

Upon binding to PAM via residues R1333 and R1335, Cas9 undergoes a conformational change that facilitates R-loop formation at the 3′ end of the crRNA. The invading strand is then unwound unidirectionally by a rotation of a phosphodiester linkage, destabilising the target DNA [100,101]. A phosphate-blocking loop then stabilises the DNA strands, maintaining continuous interaction with the crRNA, which triggers Cas9 activation and subsequent cleavage [101].

The nuclease activity of Cas9 is mediated by two metal-dependent domains (Mg^2+^): HNH and RuvC-like. These domains cleave the crRNA spacer-complementary and non-complementary (displaced) R-loop strands [70,98,102]. Following DNA:crRNA hybridisation and complex formation with Cas9, the HNH domain undergoes conformational changes that activate its DNA strand cleavage capability. Furthermore, this domain allosterically modulates the RuvC-like domain, enabling its activation and subsequent non-complementary strand cleavage [103,104]. These cleavage events occur three nucleotides upstream of the PAM sequence on both strands in the 3′ direction, generating blunt-end products [98,102].

Type III CRISPR systems exhibit distinct characteristics from other types, notably in their independence from PAM sequence recognition for targeting foreign MGEs [79]. However, certain type III-B (Cmr) subtypes, such as those found in *P. furiosus*, require PAM recognition for homologous nucleic acid targeting [105]. However, the type III CRISPR system subtypes use an RNP complex similar to the cascade (III-A presents the Csm complex, and III-B and III-C the Crm complex), exhibiting endogenous nuclease activity that degrades nucleic acids in a transcription-dependent manner [106,107,108]. These systems share similarities with type I systems, as their effector proteins—specifically, the Cas10 nuclease—contain an HD domain analogous to that found in Cas3 [109].

Type III CRISPR systems employ a distinctive self/non-self discrimination mechanism utilising 8-nucleotide complementary seed sequences at the crRNA’s 5′ end, which serve as anchoring points for subsequent RNP complex assembly [110,111]. While subtype III-A predominantly targets DNA [112], subtype III-B exhibits RNA-targeting specificity [111]. Notably, in vivo studies have demonstrated that both Csm and Cmr complexes can target plasmid DNA independently of PAM sequences [108,113]. Exceptions exist within the Csm complex family, such as the type III-A subtypes in *S. thermophilus* and *T. thermophilus*, which exhibit RNA targeting capabilities [114].

The crRNA–RNP complex assembly process begins at the 5′ end of the foreign nucleic acid, whereupon the non-complementary 3′ end of the target anti-tag region interacts with multiple sites within the Csm complexes. One crucial interaction occurs at the Csm1 or Cas10 subunit cleft (Crm2 in the Crm complex), triggering conformational changes in the L1 loop through allosteric activation. This activation leads to non-specific single-stranded DNA cleavage by the arginine-rich HD domain of Cas10 [115,116]. Concurrent with this process, Csm3/Crm4 proteins execute periodic, specific RNA transcript cleavage [116]. Structural elements within Csm3 facilitate crRNA–RNA duplex kinking, enabling target RNA cleavage with precise 6-nucleotide periodicity [116].

The Cas10 Palm domains exhibit synthetase activity, generating cyclic oligoadenylate (cOA) from ATP [115]. This molecule functions as a second messenger and allosteric modulator of specific CRISPR-associated Rossmann-fold (CARF) domain-containing effector proteins. Among these, the RNase Csm6 (also known as Csx1) employs its eukaryotic and prokaryotic nucleotide-binding (HEPN) domains to execute an auxiliary nucleic acid interference mechanism, primarily targeting single-stranded RNA [117,118].

Following the endonucleolytic activity of the Csm/Crm complex subunits, these proteins undergo progressive deactivation as nucleic acid cleavage proceeds [119]. Studies have revealed that Cas6/Csx1 proteins possess cOA degradation capabilities, demonstrating a self-inhibitory regulatory mechanism. Similar inhibitory mechanisms exist in Cas10, where specific glutamine residues mediate its activity [116].

While sharing similarities with types I and III regarding effector protein utilisation for bacterial adaptive immunity, type IV CRISPR systems exhibit distinct functional characteristics [68]. These systems actively counter plasmid propagation mechanisms and enhance nucleic acid recombination events [120,121]. However, the complete mechanistic details of type IV systems remain to be fully elucidated.

Current hypotheses regarding type IV systems’ plasmid targeting mechanisms highlight the role of a putative helicase (DinG/RecD) in subtype IV-A, which is essential for plasmid target specificity [68]. This helicase demonstrates mechanistic similarities to the type I system’s Cas3 [122], and its interaction with target strands may promote destabilisation, thereby enhancing the bacterial protective mechanisms of type IV systems [68].

Type V CRISPR systems parallel type II systems in utilising a single effector protein for targeting invading nucleic acids. Cas12 is the primary interference mechanism in this system across subtypes V-A, V-B, and V-C (corresponding to Cas12a, Cas12b, and Cas12c, respectively) [47]. The protein recognises the seed region of the target dsDNA PAM and executes cleavage of both strands through its RuvC-like domain [123,124]. Unlike subtype V-B, Cas12a functions independently of tracrRNA for MGE recognition and destruction, requiring calcium or magnesium ions for activity [73]. The V-F subtype employs Cas12f (Cas14) specifically for ssDNA cleavage [125].

Type VI systems exhibit a distinctive characteristic among class 2 CRISPR systems: beyond targeting crRNA-homologous invading nucleic acids, they also perform non-specific RNA degradation [125]. The crRNA–Cas13 complex recognises a PAM sequence at the 3′ end of single-stranded RNAs, triggering conformational changes in Cas13 that initiate RNA cleavage, preferentially at uridine-rich sites [80,126]. Despite the diverse array of Cas proteins and intermediates involved in the adaptive immunity mechanisms across CRISPR systems, they converge on a common pathway: recognition of foreign MGEs and hybridisation and/or interaction between crRNA and homologous nucleic acids, culminating in target destruction.

## 3. CRISPR-Cas Delivery Systems

One of the most prominent challenges CRISPR-Cas-based therapy faces is the proper delivery of the cargo into the target cells. The CRISPR-Cas system can be delivered in three different forms. The first alternative is an sgRNA and mRNA of Cas9 protein, which can be inserted into the cytoplasm, and the mRNA is subsequently translated. However, the stability of the mRNA molecule is poor, causing its fast degradation and, therefore, limited gene editing duration. Another cargo option entails the delivery of a plasmid DNA encoding Cas9 and sgRNA. This alternative has been demonstrated to be more stable, though less effective, since the plasmid needs to be within the cell nucleus. The third form is the Cas9–sgRNA RNP complex, an effective and safe alternative limited by molecular weight and active Cas9 synthesis in large quantities [5,127].

Various systems to deliver these cargos have been developed and tested. The strategies can be classified into two main categories: viral and non-viral delivery systems [128].

### 3.1. Non-Viral Delivery Strategies

Within non-viral options, physical methods and nanoparticles represent the main sub-categories. Unlike viral delivery systems, these alternatives have been proven efficient and do not limit the cargo size. However, tissue damage and cell viability have proven to be a considerable obstacle, particularly for physical delivery methods [129]. Here, we list some of the most relevant strategies for non-viral delivery, including their pros and limitations.

#### 3.1.1. Microinjections

Microinjections consist of using a microinjection needle within the visual range of a microscope to insert the cargo into the target cells individually. This method directly delivers different cargos, such as DNA plasmids, mRNA, or cas9 proteins with sgRNA. Consequently, this technique has proven simple, and the cargo material can be transported independently of molecular weight with minimal off-target side effects [127].

However, it is worth pointing out that the straightforward nature of the procedure allows for only a few cells to be inserted simultaneously. Though harmless to other tissues, the microinjections may cause cellular damage at the injection site. Lastly, this strategy is more suited to in vitro work due to the need for a microscope to insert the material. Recent investigations using microinjections to deliver CRISPR-Cas in different living organisms have rendered positive results. A wide range of experimental trials using arthropods, mice, and even human embryos have been carried out successfully; however, the effectiveness of microinjections in many other living organisms has not been thoroughly explored [130].

In this regard, the first successful study in plant cells was performed by Szabata et al. in 2024 using CRISPR-Cas9 binary vectors to target the *Ms2* gene of wheat microspores via microinjection, resulting in successful deletions of 1–16 bp of the target gene [131]. Furthermore, Ran Li et al. applied microinjections to deliver CRISPR-Cas9 system into *Neocaridina heteropoda* embryos. By this method, the Nh-scarlet gene was successfully knocked out, contributing to future functional genomic studies in other crustaceans and proving the versatility and effectiveness of microinjections in diverse living organisms [132].

#### 3.1.2. Electroporation

Another widespread physical method is the transient disruption of the phospholipid bilayer of the plasmatic membrane through controlled electric pulses in order to increment cell membrane permeability, allowing intracellular transport and, therefore, the introduction of nucleic acids into specific cells, a process called electroporation [133]. This method is suitable for all types of CRISPR-Cas cargo and contrasts with other popular delivery systems, such as microinjections, for various reasons. Firstly, since a microscope is not required to perform the delivery, this method can be used both in vitro and in vivo. In addition, higher survival rates in embryos have been reported [129].

Nonetheless, electroporation is not free of downsides, such as requiring very specific cell conditions, such as the electric field features, electrode geometry, and cell and cargo types, which could lead to higher costs. Furthermore, issues such as cell death are to be considered, particularly in stress-sensitive cells, and the position of the cell in relation to the electrodes, making a heterogeneous overall response to the procedure. Researchers have recently improved the traditional approach to electroporation to circumvent some of the aforementioned obstacles. Huaigeng et al. developed a protocol using human pluripotent stem cells (PSCs) and ribonucleoprotein (RNP) of CRISPR-Cas9 as cargo to optimise issues such as inefficient delivery and prolonged duration. The insertion site was proposed to be modified, and MaxCyte and 4D nucleofector electroporators utilised, resulting in successful gene editing. However, this protocol is limited to proliferating cells with a specific PAM sequence [134].

#### 3.1.3. Hydrodynamic Delivery

Hydrodynamic delivery uses a rapid injection of fluid, generally saline solution, into the tail vein of the animal, with a volume of 8–10% of its total body weight. This liquid is loaded with gene editing cargo into the animal’s bloodstream; the quick flow of saline solution produces liver expansion, increasing hydrodynamic pressure and temporarily disrupting the endothelium and cell membranes, therefore permitting the passage of CRISPR-Cas cargo into the cells, particularly hepatocytes and the cells of the kidneys, lung, muscles, and heart. This in vivo technique, although attractive for its simplicity, is not currently considered a plausible procedure in humans since the injection of large volumes of fluids has been shown to accumulate on the inferior vena cava, increase blood pressure, and cause temporary cardiac dysfunction, liver expansion, and even death of the subject [135].

### 3.2. Viral Delivery Strategies

Viruses currently represent one of the most utilised alternatives regarding CRISPR-Cas delivery. Viruses such as adenoviruses (AdVs), adeno-associated viruses (AAVs), and lentivirus stand out among the main options. Despite being popular alternatives, viral systems pose a few conditions; for example, viruses require HEK 293T cells to synthesise viral-like particles containing Cas9 and sgRNA. These particles invade the target cells and are posteriorly inoculated into the desired organism or studied in vitro [136]

#### 3.2.1. Adenoviral Vectors

Adenoviruses are double-stranded DNA viruses that can affect dividing and non-dividing cells. Certain properties of these types of viruses allow the effective delivery of genetic material after inoculation; the virus genome does not integrate with the host genome, diminishing side effects like insertional mutagenesis. Due to this, their transduction effectiveness, and low immunogenicity, AdVs constitute one of the main candidates for viral delivery systems [137]. Nevertheless, these vectors present some limitations. On the one hand, AdVs are small, and therefore, the size of the cargo is restricted; to solve this issue, researchers have proposed to package different parts of the genetic material, such as sgRNA and Cas9 proteins, in separate vectors and inoculate them simultaneously [138].

#### 3.2.2. Adeno-Associated Viruses

As AdVs, these vectors are DNA double-stranded viruses that can infect both dividing and non-dividing cells; as one of the most popular options, they possess an adequate safety profile with mild immunogenicity and off-target side effects. These viruses do not insert their genome into the host chromosomes. Instead, the viral genetic material binds into specific loci of mitochondrial DNA known as integration sites, and this mechanism maintains the safety profile of AAVs since it does not contribute to tumorigenesis [139].

An important limitation of this delivery system is the limited capacity of cloning attributed to these vectors, which translates into a limited payload and difficulties in packaging the genetic material; to solve this issue, researchers have used dual or triple approaches or smaller Cas9 orthologs. In addition to their defective replication, AAVs present other limitations, such as potential cytotoxicity and isolated mutations [140].

#### 3.2.3. Lentivirus

On the other hand, lentivirus is a single-stranded RNA virus able to act in dividing and non-dividing cells with powerful cloning and cargo capacities. Unlike the previously mentioned vectors, one of its distinctive features consists of its ability to be pseudotyped with other viral proteins; in addition, the immunogenicity of this vector is quite low, and they possess good packaging and transduction potential as well as minimal effects on the cell cycle. However, unlike AdVs, lentiviruses integrate their genome into the host’s genetic material, potentially leading to insertional mutagenesis and other off-target side effects [141].

Investigators have proposed alternatives such as integrating defective lentiviruses (IDLVs) to mitigate the potential side effects. In this approach, a single point mutation is introduced to an integrase, hampering lentivirus integration and augmenting the safety of LV vectors; however, on the downside, IDLVs show lower transgene expression. Moreover, another feasible alternative to reduce the mutational risk is using a self-inactivating Cas9 protein to reduce Cas9 off-target adverse effects [142].

## 4. Recent Discoveries: Where Are We Now?

The CRISPR system’s unique characteristics have undergone extensive investigation, revealing its potential and limitations in medical and clinical applications. This revolutionary technology has catalysed unprecedented research momentum and continues to generate new questions and possibilities for human applications [143,144]. Rarely has such rapid progress been observed in deepening knowledge in the scientific community, but the call of biotechnology has always been incentive enough to drive the search for answers.

It is due to this constant search for improvement and precision in favour of the ease of use of tools such as CRISPR-Cas that have led to tentative discoveries that could expedite genetic editing in previously studied cases.

### 4.1. Functional and Chemical Modifications of RNA

In recent years, several advances have been made in the functional and chemical modification of the RNA of CRISPR systems to improve editing efficiency, reduce off-target effects, and provide unique functionalities [145,146]. In this vein, in CRISPR-Cas9 systems (type II), a certain chain of events is necessary for the enzymatic function to properly cleave the target DNA sequences in question, with the presence of both crRNA and tracrRNA bound being imperative to fulfil its endonuclease function [147].

TracrRNA is strictly necessary for pre-crRNA maturation; consequently, Cas9 cannot be properly activated in its absence. This “pre-requisite” could make the process of preparing RNA guides more tedious and prone to errors in research laboratories, which is why the idea of synthesising a hybrid or chimeric RNA guide that combined crRNA and tracrRNA in a single RNA strand, called sgRNA, arose [102,148]. This arrangement mimics the natural structure of the crRNA–tracrRNA pair and was designed to simplify the process of gene editing; however, it has been determined that both types are equally efficient, and only a few specific situations have been proposed in which sgRNA could be the better option (for example, in cases where the complex would be exposed to a high concentration of cellular exo- and endonucleases) [149].

Chemical modifications are mainly based on long RNA sequence synthesis with modified phosphoramidites and 2′-F and 2′-OMe monomers and ribonucleotide exchange for DNA homologs. Concerning the latter, it has been shown that the replacement of 5′-terminal ribonucleotides with crRNA deoxyribonucleotides preserves DNA cleavage activity using SpCas9 in addition to significantly reducing the off-target activity of crRNA; however, the addition of 3′-terminal deoxyribonucleotides may serve as another tool to improve crRNA design [150,151,152].

Concerning the modification of 3′ and 5′ terminal nucleotides by 2′-F and 2′-OMe monomers, it is useful in improving Watson–Crick H-binding and in the metabolic stabilisation of crRNA, optimising its activity; results are similar to those obtained when modifying the 2′-OH groups of crRNA with 5-carboxylcytosine [153,154]. The 2′-F modifications are also well tolerated in the distal PAM region of the crRNA spacer, which is associated with greater stability of the crRNA–tracrRNA complex and lower off-target activity [155].

Phosphate backbone modifications have also been shown to be useful for Cas9 enhancement [156]. In particular, 2′-O-methyl 3′-phosphonoacetate (2′-O-Me 3′-PACE) and 2′-O-methyl 3′-thiophosphonoacetate (2′-O-Me 3′-thiophosphonoacetate) are listed as two crRNA backbone modifications that improve crRNA stability and bioavailability and increase the activity and efficiency of editing [155,157,158]. In turn, crRNAs were modified using two strategies in combination, phosphate backbone modification and sugar addition, which has been shown to improve Cas9 DNA cleavage and help avoid toxicity in U2OS and HeLa cell models [159].

On the other hand, in vitro models have shown that modification of uridine nucleosides in tracRNA and crRNA by enzymatic modification and nanoparticle technology can enhance Cas9 activity and its ability to elicit an immune response [160]. Undoubtedly, the field of RNA metabolic modification with CRISPR-Cas systems is constantly being rediscovered and developed, allowing it to be improved every day as an unprecedented gene editing tool. It is important to carry out further studies on the metabolic modification of other CRISPR-Cas systems in addition to Cas9.

### 4.2. Base Editing

A significant advancement in Cas9 applications involves precise base editing capabilities. While traditional CRISPR-Cas9 operates through double-strand DNA cleavage followed by cellular DNA repair mechanisms that result in coding sequence knockout preceding the PAM [161], researchers have engineered modified versions (dCas9 or nCas9) that retain DNA targeting ability while lacking enzymatic activity [162].

This innovation led to the development of fusion proteins combining dCas9/nCas9’s targeting capability with novel functionalities, enabling precise base substitutions and genetic regulation [163]. These base editors integrate programmable deaminases with catalytically inactive Cas9, yielding adenine base editors (ABEs) and cytidine base editors (CBEs). These systems catalyse the hydrolysis of amino groups in A and C, respectively, facilitating adenosine-to-guanosine and cytosine-to-thymine conversions [164].

ABE8s represents the state-of-the-art adenine base editing, surpassing its predecessor, ABE7.10, in precision [165]. For cytosine base editing, BE4 has demonstrated superior performance in experimental studies [166]. While both editor types have been optimised for specificity and efficiency, off-target effects remain a significant challenge. Recent research has identified potential solutions, including anti-CRISPR proteins AcrIIA4 and AcrIIA5, which effectively inhibit CRISPR activity in human cells, offer a promising approach to mitigate off-target modifications [167].

### 4.3. Base Prime Editors

Prime editors represent an evolution in precise genetic modification, building upon the concept of using Cas9 as a targeting mechanism without full catalytic functionality. This system employs a modified Cas9 retaining only RuvC domain activity, executing single-strand DNA cleavage rather than double-strand breaks, thus preventing immediate cellular repair responses [168]. The system combines this modified Cas9 with a reverse transcriptase (RT), guided by a prime editing guide RNA (pegRNA) that incorporates multiple functional elements: a DNA-targeting sequence, tracrRNA sequence, RT primer binding site, and a template containing the desired genetic modification [169].

Researchers have developed four prime editor variants, each demonstrating varying efficiencies in human cell studies [141]. Prime editors offer significant advantages over conventional base editors, notably, reduced off-target effects and the capacity to execute all twelve possible base conversions, compared with the four transition mutations (C > T, G > A, A > G, and T > C) possible with standard base editors. This expanded capability has implications for treating various genetic disorders, including Tay–Sachs disease, certain forms of sickle cell anaemia, and cystic fibrosis [170].

Base-prime editors in in vivo applications for genetic diseases remain a challenge. In the following study, a reduced-size SpCas9 primer editor without the RNAse H domain and an integrin cleavage construct was developed using liver-associated adenovirus delivery, which achieved 15% efficiency at the Dnmt1 locus, increasing by 58% via PE2ΔRnH delivery without the cleavage, using the human adenoviral vector 5. The trial to correct a genetic liver disease using the AdV approach to repair the Pahenu2 mutation causing phenylketonuria (PKU) resulted in an average deficiency of 11.1% [171]. Another case corresponds to the use of adenine bases for the correction of Duchenne muscular dystrophy (DMD) to modify the splice donor sites of the dystrophin gene, causing skipping of a common DMD deletion mutation of exon 51 by the associated adenovirus serotype 9 that modifies adenine bases, restoring dystrophin expression in human pluripotent stem cell-derived cardiomyocytes. The above results demonstrate the efficacy of prime editors for correcting various DMD or PKU mutations, but limitations can be observed regarding improved delivery methods or higher doses of administration [172].

### 4.4. Gene Regulation

The catalytic inactivation and protein hybridisation of Cas9 have opened new avenues in gene regulation and epigenetic modification. Eliminating Cas9’s enzymatic activity while preserving its DNA targeting capability creates mechanical interference with transcription processes, effectively blocking RNA polymerase activity. This interference can prevent transcription factor assembly, depending on the dCas9 binding site, resulting in targeted gene expression silencing [173].

Further developments have produced regulatory complexes through the fusion of dCas9 with transcriptional effector domains, creating activation (CRISPRa) or inhibition (CRISPRi) systems for gene expression control [174]. A key technical consideration is a requirement for sgRNA design targeting promoter regions or transcription start sites for effective gene modulation. This process can present challenges when these regions are poorly characterised or inaccessible due to local molecular factors. Nevertheless, CRISPRi/CRISPRa systems have demonstrated significant utility in cellular signalling studies and cancer-related gene screening investigations [175,176].

In the epigenetic domain, dCas9 fusion with histone modifiers or DNA methylation enzymes enables targeted epigenetic modification. For instance, combining dCas9 with the DNMT3A catalytic domain, essential for de novo methylation, enables site-specific DNA methylation. Current research focuses on optimising methyltransferase combinations to enhance specificity and reduce off-target effects [177].

Conversely, dCas9 fusion with the TET hydroxylase catalytic domain creates targeted demethylation capabilities. Both in vitro and in vivo studies have demonstrated the utility of this approach in investigating disease models associated with methylation abnormalities, suggesting potential therapeutic applications [178].

### 4.5. CHyMErA

**Figure 3:**
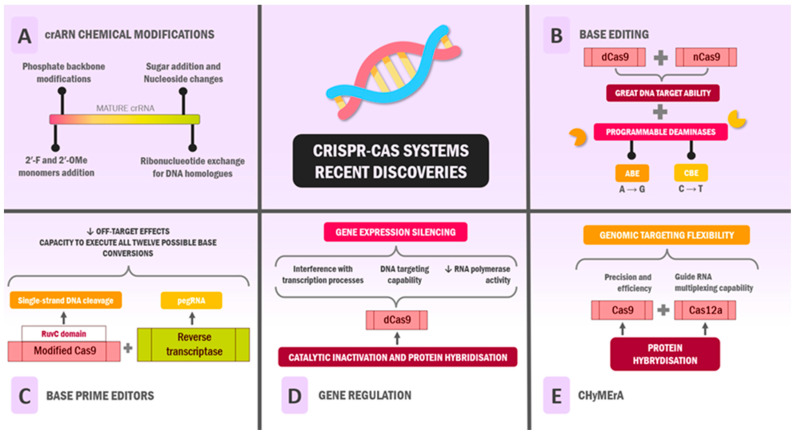
CRISPR-Cas systems recent discoveries. In recent years, the scientific community has been searching for new tools to improve the properties of CRISPR-Cas systems. These include (**A**) crRNA chemical modifications; (**B**) base editing; (**C**) base prime editors; (**D**) gene regulation; (**E**) ChyMErA. Abbreviations: DNA: deoxyribonucleic acid; RNA: ribonucleic acid; CHyMErA: Cas Hybrid for Multiplexed Editing and Screening Applications; pegRNA: prime editing guide RNA; ABE: adenine base editor; CBE: cytidine base editor.

The exploration of protein hybridisation has led to the development of CHyMErA (Cas Hybrid for Multiplexed Editing and Screening Applications), which combines the functionalities of Cas9 and Cas12a. This hybrid system leverages Cas9’s precision and efficiency while incorporating Cas12a’s guide RNA multiplexing capability, enhancing genomic targeting flexibility. A notable achievement of CHyMErA has been the successful targeting of multiple cancer-associated exons that were previously inaccessible to Cas9 alone [179]. See Figure 3.

Moreover, other useful combinatorial strategies have been recently developed; in a study by Pacalin et al., a new system, CRISPRai, was introduced. This novel strategy involves activating (CRISPRa) and repressive (CRISPRi) alterations into two loci simultaneously within the same cell to study genetic perturbations in mixed-cell populations. This study analysed the genetic interaction of SPI1 and GATA1 and the IL-2 effects on CART cells and Jurkat T cells, concluding that this combinatorial technique could give more insight into genetic interactions [180].

### 4.6. CRISPR Screening

In recent years, novel approaches such as CRISPR screening have been proposed. This technique is based on a large-scale application of the CRISPR-Cas systems that leads to the modification of genetic material in multiple cells simultaneously, with the object of identifying certain features or biological processes [181].

This method can be executed in different forms; classically, CRISPR screening creates genetic knockouts or loss of function. When this mechanism is combined with deep-sequencing techniques and effectuated on a large scale, multiple genomic elements stand out as candidates [182]. On the other hand, pooled CRISPR screens is another method that consists of the introduction of certain genetic alterations into a pool of cells; the cellular population proliferates under a specific biological condition (drug treatments, viruses, cellular competition, among others), and the consequence of the genetic modification is then assessed by genome sequencing, resulting in a list of genes that potentially contribute to either resistance or sensitivity to the specific biological condition [183].

These approaches have constituted a novel source of research on gene editing, and their implications could extend to different fields, from discovering new drugs to unravelling the complex pathophysiological basis of multiple diseases.

### 4.7. Anti-CRISPR Proteins

Recent research has identified potential solutions, including the anti-CRISPR proteins AcrIIA4 and AcrIIA5, which effectively inhibit CRISPR activity in human cells, offering a promising approach to mitigate off-target modifications [167]. While anti-CRISPR proteins were first discovered in experiments with P. aeruginosa, bioinformatics tools have recently enabled new anti-CRISPR proteins, with at least 21 such protein families now available [184,185].

Within the mechanism of action of the anti-CRISPR proteins, the most important are interfering with the loading of crRNA, preventing the activity of Cas9 (AcrIIC2 family) and preventing the excision of specific DNA by binding to subunits of the Cas–Cas effector complex of the CRISPR-Cas system (AcrIF family), promoting the dimerisation of Cas9 and mimicking the PAM recognition residues (AcrIIA2) [186,187,188,189]. Still, the mechanisms of some types of anti-CRISPR proteins have not been elucidated. However, bacteriophage technology has allowed progress for anti-CRISPR proteins in human cells. In particular, AcrIIA1 binds to the HNH domain of Cas9 and induces the degradation of SpyCas9 and SauCas9 in human cells, which may modulate the activity of these nucleases and thus reduce the off-target effects of CRISPR use in medicine [190].

## 5. Integrate

Recent developments have addressed a fundamental limitation of CRISPR-Cas systems: their dependence on cellular DNA repair mechanisms, which can lead to unintended deletions and mutations. Researchers have introduced the INTEGRATE tool, a self-contained DNA editing system derived from Vibrio cholerae, that functions independently of cellular repair mechanisms. This CRISPR-transposon system enables site-specific DNA integration through RNA-guided integrases. While initial validation has been successful in bacterial genomes, ongoing research aims to extend the applications to mammalian cells [191,192].

### 5.1. PAM Variability

A significant constraint in CRISPR-Cas applications has been the limited range of PAM sequences recognised by various Cas proteins, with traditional Cas9 primarily recognising 5′-NGG-3′ PAMs (where N represents any nucleotide) [193,194]. Recent research has focused on engineering Cas9 variants with expanded PAM recognition capabilities [195,196].

An example of this is Cas9 mutant versions such as xCas9-NG (a hybrid resulting from the fusion of xCas9 and Cas-NG, both Cas9 variations with PAM flexibilisation) [197], which, although it has reported markedly less activity than traditional Cas9, also greatly expands the system’s field of action of CRISPR [198]. These modifications provide versatility to the editing tool that could lead to a greater number of research and clinical applications.

### 5.2. Mini CRISPRs

The molecular size of CRISPR complexes has presented challenges for delivery systems, as larger dimensions limit the number of molecules that can be effectively transported via various vectors. Recent innovations have produced ultra-compact CRISPR systems, known as CasMINIs, engineered from the Cas12f protein of the V-F system combined with an optimised single RNA chain (sgRNA). This system achieves approximately 50% size reduction compared with conventional systems, potentially expanding CRISPR applications through improved delivery efficiency [199,200].

Furthermore, other mini-Cas proteins have proven useful in genome engineering; the SpCas9 derived from Staphylococcus aureus can recognise specific PAM sequences to induce mutations in different plants, from rice to citrus and tobacco [201]. Moreover, CjCas9, derived from *Campylobacter jejuni,* is an even smaller alternative, which allows these systems to be delivered using AAV systems. Similarly, CasX is an RNA-guided endonuclease with two homologue systems from *Deltaproteobacteria* and *Planctomycetes* that belongs to the miniCRISPRs group; however, some differences can be pointed out between this miniCas and other compact proteins; notably, this protein is small enough to fit into a single adenovirus delivery system with additional room for other complexes. However, the activity of CasX is less potent than that of Cas12f or the standard Cas9 [199]. Lastly, CasΦ is an ultra-compact system that comprises a crRNA active site for processing and crRNA-guided DNA cutting target for other nucleic acids. This system has been studied in vitro and in vivo, concluding that this miniCas could be a potential tool for DNA deletions for half the molecular weight of a standard CRISPR system [202].

## 6. Conclusions

The CRISPR-Cas system’s mechanism derives from microbial adaptive immunity processes, comprising three theoretically consistent stages that vary primarily in their associated Cas proteins. However, our understanding of these systems remains incomplete. The extensive functional and structural variations in CRISPR loci and their associated proteins continue to yield discoveries. Further research is essential to understand these systems comprehensively, clarify existing component mechanisms, and advance CRISPR-Cas development as a standard therapeutic tool for genetic disorders.

## Abbreviations

CRISPR	Clustered Regularly Interspaced Short Palindromic Repeats
HIV	Human Immunodeficiency Virus
TALEN	Transcription Activator-Like Effector Nuclease
ZFN	Zinc Finger Nuclease
DNA	Desoxyribonucleic Acid
SRSR	Regularly Spaced Short Repetitions
RNA	Ribonucleic Acid
crRNA	CRISPR RNA
NUC Lobe	Nuclease Lobe
REC	Recognition Lobe
RNP	Ribonucleoprotein Particle
tracrRNA	Trans-Activating crRNA
CTD	C-terminal Domain
PAM	Protospacer Adjacent Motif
RRM	RNA Recognition Motif
RAMP	Receptor Activity-Modifying Protein
RAMPs	Repeat-Associated Mysterious Proteins
mRNA	Messenger RNA
PAPS	3′-Phosphoadenosine-5′-phosphosulfate
PI Domain	PAM Interacting Domain
BH	Bridge Helix
dsDNA	Double Strand DNA
HEPN Domain	Higher Eukaryotes and Prokaryotes Nucleotide-Binding Domain
NTD	N-Terminal Domain
RHH	Ribbon-Helix-Helix
HTH	Helix-Turn-Helix Domain
MGE	Mobile Genome Expression
ssDNA	Single-Stranded DNA
IHF	Integration-Host Factor
ATP	Adenosine Triphosphate
ABE	Adenine Base Editor
CBE	Cytidine Base Editor
pegRNA	Prime Editing Guide RNA
CRISPRi	CRISPR Interference
CRISPRa	CRISPR Activation
DNMT3A	DNA Methyltransferase 3 Alpha
TET	Ten-Eleven Translocation
ChyMeRa	Cas Hybrid for Multiplexed Editing and Screening Application
